# Intraosseous Lipoma of the Calcaneum

**DOI:** 10.7759/cureus.16929

**Published:** 2021-08-06

**Authors:** Pankaj Kumar Sharma, Zile Singh Kundu, Vivek Tiwari, Vijay Kumar Digge, Jyoti Sharma

**Affiliations:** 1 Orthopaedics, All India Institute of Medical Sciences, Bathinda, IND; 2 Orthopaedics, Positron Multi-Speciality and Cancer Hospital, Rohtak, IND; 3 Orthopaedics, All India Institute of Medical Sciences, Nagpur, IND; 4 Orthopaedics, All India Institute of Medical Sciences, New Delhi, IND; 5 Anaesthesiology, All India Institute of Medical Sciences, Bathinda, IND

**Keywords:** lipoma, benign tumor, bone tumor, calcaneum, heel pain

## Abstract

Lipomas are benign lesions of adipose tissue, which commonly affect the soft tissues but are rarely found in the musculoskeletal system. Intraosseous lipomas are rare benign tumors and even rarer in calcaneum, only to be found incidentally in the majority of cases. We report a case of a 45-year-old male patient who presented to the outpatient department with complaints of bilateral heel pain, which was initially treated conservatively as the presentation was similar to plantar fasciitis. On further follow-up, a plain radiograph of the ankles was taken, which showed a lytic lesion of the calcaneum with mild sclerotic margins on the right side with normal left foot radiographs. On magnetic resonance imaging, the lytic lesion demonstrated hyperintense signals on T1-weighted sagittal images, characteristic of fatty tissue, which helped us in arriving at the diagnosis of an intraosseous lipoma. The patient was treated by conservative means with physiotherapy, which relieved the pain, and on serial follow-ups, the lesion was found non-progressive on successive radiological evaluation. The differential diagnosis of such an entity includes plantar fasciitis, tumors such as an aneurysmal bone cyst, bone infarct, etc. With the increasing use of magnetic resonance imaging and computed tomography scans, physicians should be aware of the possibility of an intraosseous lipoma of the calcaneum, which should be ruled out during evaluation. Although possible, malignant pathology or aggressive transformation of such lesions is very rare; however, the lesion should be evaluated adequately and managed by surgical means in cases non-responsive to various conservative modalities.

## Introduction

Lipomas are benign lesions of adipose tissue and affect soft tissues, including the musculoskeletal system [1]. However, lipomas are not commonly found inside bones. Moreover, intraosseous lipoma of the calcaneum is one of the rare occurrences and is often misdiagnosed. The differential diagnosis of such an entity includes plantar fasciitis, tumors such as an aneurysmal bone cyst, bone infarct, etc. [1-3]. A careful evaluation with plain radiographs, magnetic resonance imaging (MRI), computed tomography (CT), and histopathological correlation is needed to avoid misinterpretation [3-5]. We report a case of intraosseous lipoma of the calcaneum in a 45-year-old male patient who was initially treated as plantar fasciitis based on the symptoms of heel pain. A review of literature of similar cases in English literature is also done in this report.

## Case presentation

**Figure 1:**
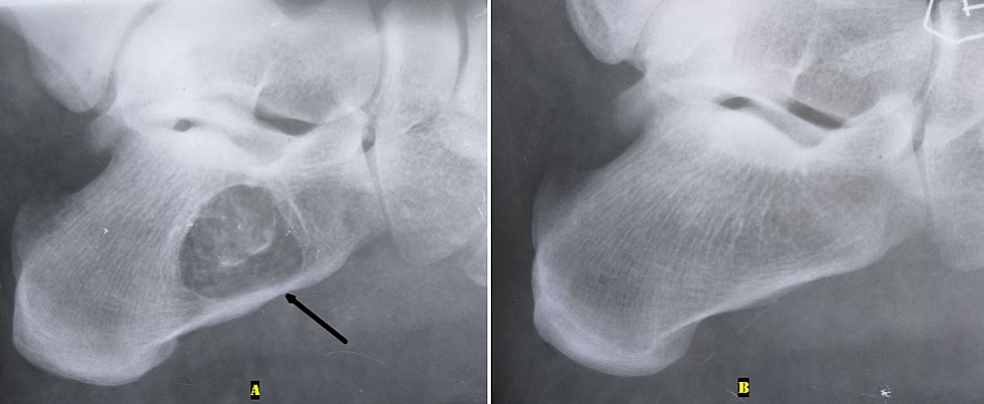
Plain radiograph of both ankles' lateral view A: A lytic lesion was seen in the body of the right intact calcaneum with mild sclerotic margins (black arrow). B: A normal left calcaneum.

A 45-year-old male patient, a laborer by occupation, presented to the outpatient department with bilateral heel pain for the past three months, which was aggravated on weight-bearing. There was no history of significant trauma or similar kind of illness in the past. He was an occasional alcoholic and smoker for the past 10 years. On examination, there was no swelling, deformity, or any inflammatory/trophic changes. Tenderness was present over bilateral heels, more on the right side, and clinically plantar fasciitis was suspected. No palpable mass or bony swelling was appreciated on palpation. Plantar flexion and dorsiflexion movements were normal, while inversion and eversion movements were terminally painful on the right foot. Previously, the patient was diagnosed and treated for plantar fasciitis with analgesics, hot fomentation, and shoe modification, where he got relieved symptomatically in the left foot. All the routine hematological and systemic investigations were normal. A plain radiograph of both ankles' lateral view revealed a lytic lesion in the body of the right calcaneum measuring around 3 x 2.5 cm with mild sclerotic margins, while the left side was found to be normal (Figure 1).

**Figure 2:**
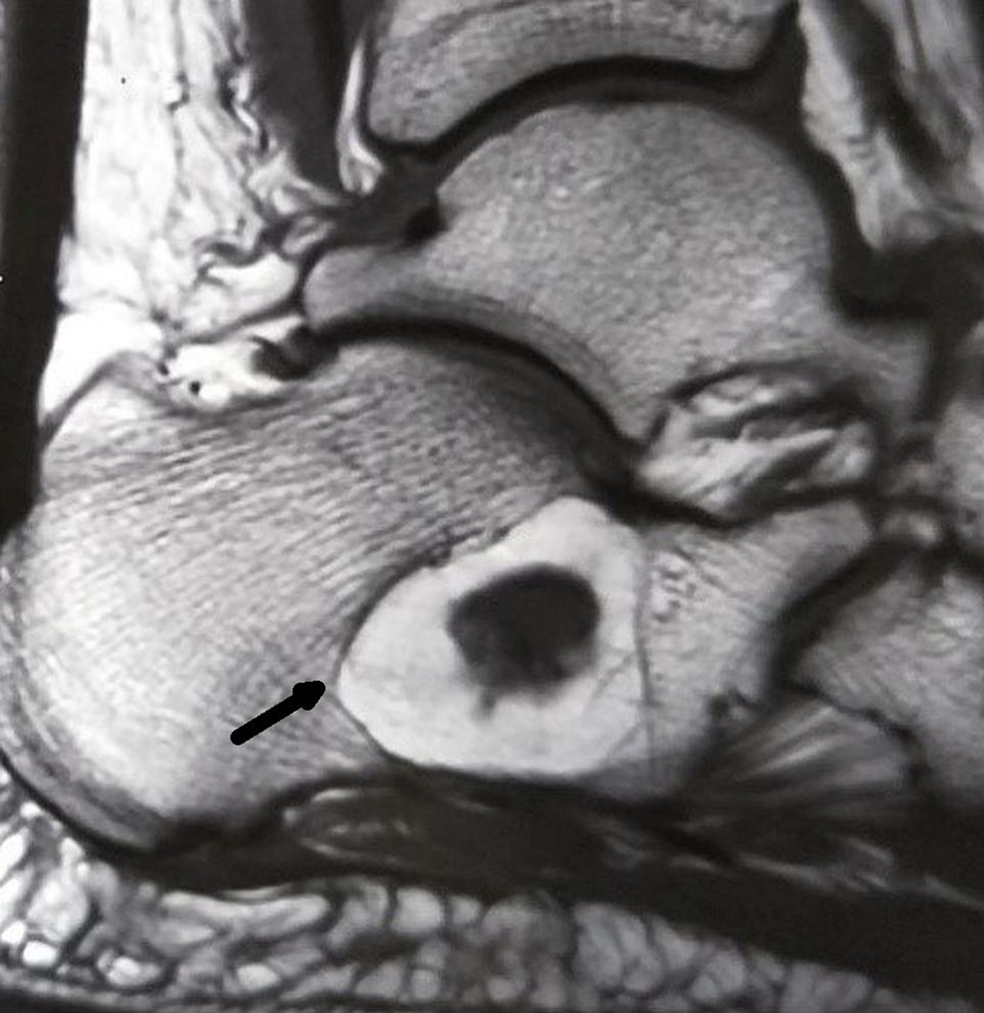
Magnetic resonance imaging of the right ankle at presentation The T1-weighted sagittal image demonstrated a 3 x 2.5 cm lytic lesion in the body of calcaneum (black arrow), with hyperintense signals characteristic of fatty tissue, in addition to minimal effusion in the plantar fascia and an intact calcaneum.

An MRI of the right ankle was sought, which showed a lesion in the calcaneum with hyperintense signals on T1-weighted sagittal images, which is characteristic of fatty tissue, in addition to minimal effusion in the plantar fascia (Figure 2).

**Figure 3:**
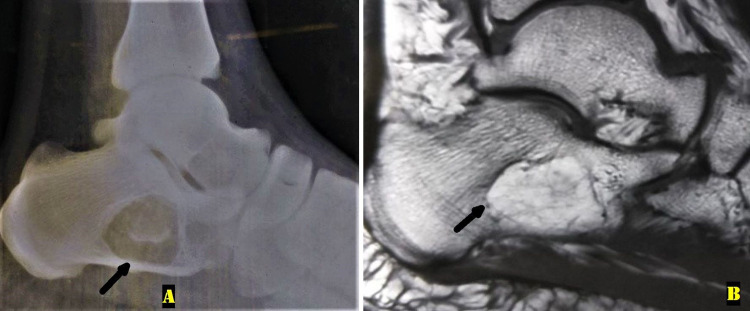
Radiologic evaluation at three years follow-up A: Plain radiograph of the right ankle lateral view showing no progression in the lytic lesion with an intact calcaneum (black arrow). B: A T1-weighted sagittal magnetic resonance image showed a non-progressive fatty lesion in the right calcaneum (black arrow), similar to the first presentation.

Thus, the diagnosis of intraosseous lipoma was made clinico-radiologically, and the patient was informed regarding the rare incidence and the mode of treatment in the form of conservative or surgical intervention. The patient opted for conservative management under observation, and his symptoms were relieved with analgesics and hot fomentation. On the third serial follow-up, i.e., three months following the initial diagnosis, the patient was completely symptom-free. There was no relapse at the final follow-up of three years with no radiologic progression in the lytic lesion (Figure 3).

## Discussion

Intraosseous lipomas are benign tumors with an incidence of 0.1% of all bone tumors. These tumors mostly affect bones of lower limbs (71%), with calcaneum accounting for around 32% of all such cases [3]. These are derived from mature adipocytes, but there is a lack of uniform consensus regarding the etiology of this entity [1]. At present, most of the literature favors its primary origin from bone marrow fat, but theories of trauma and fat degeneration, infective pathologies, and fat metaplasia were also documented in the past. There is no age and gender predilection, but some studies documented them to be common in the fourth decade of life with slight male affection [2]. The diagnosis is usually established as an incidental finding of a lytic lesion on radiographs with symptoms of minor aching/discomfort in the heel [1]. The most common clinical feature is non-specific heel pain, while pathological fracture of the calcaneum has also been observed in extremely rare cases [6,7]. The initial diagnosis can be confirmed by CT scans or more specifically with MRI with hyperintense signals on T1-weighted images, suggestive of fatty tissue. These lesions can be classified according to their origin and location into intramedullary, central, cortical, and superficial (periosteal) [4]. These can also be staged, as described by Milgram, based on their radio-pathological findings into stages 1-3 where stage 1 shows minimal replacement of trabecular pattern of bone with fat and adipocytes, and stage 3 comprises of resorption of parent bone within lesion with complete lipoma formation by mature lipocytes [5,8]. Usually, confirmation with histopathology is not required, and a final histopathological examination is done for the excised specimen during surgery. These lesions have very slow progression and are benign in nature, so they usually improve with conservative treatment with a good prognosis. There is no documentation of malignant transformation in calcaneal intraosseous lipoma, but the possibility of malignancy may be suspected in long bone lesions in chronic cases if rapid destruction is seen [9]. There are several documented reports in the literature, which support its presentation and management (Table 1).

**Table 1:** Documented reports in the literature regarding intraosseous lipoma revealing the nature of the entity and management by different authors

Literature	Study titles	Comment and conclusions
Milgram (1988) [1]	Intraosseous lipomas. A clinico-pathologic study of 66 cases	Involution-based staging: stage 1 (tumors of viable fat cells), stage 2 (transitional cases composed of partial fat necrosis and calcification), and stage 3 (includes cyst formation).
Narang and Gangopadhyay (2011) [2]	Calcaneal intraosseous lipoma: a case report and review of the literature	Diagnosed by CT scan and histopathological analysis and treated by curettage and calcium phosphate bone graft substitute.
Campbell et al. (2003) [3]	Intraosseous lipoma: report of 35 new cases and a review of the literature	Management mostly involves recognition of bone lesion as an intraosseous lipoma, which may be regarded as an endpoint to an investigation, while biopsy and surgery can be avoided in the majority.
Milgram (1988) [5]	Intraosseous lipoma: radiologic and pathologic manifestations	Malignant transformation of a lipoma should be suspected when rapid bone destruction is seen in stage 1 (radiolucent lipoma). Stage 3 lipomas can be mistaken for malignant transformation within bone infarcts.
Csizy et al. (2001) [6]	Benign calcaneal bone cyst and pathologic fracture- surgical treatment with injectable calcium-phosphate bone cement (Norian): a case report	Percutaneous technique included a minimally invasive option for treatment of a calcaneal bone cyst with pathologic fracture.
Karthik and Aarthi (2011) [10]	Intraosseous lipoma of the calcaneus mimicking plantar fascitis	Radiographs should be indicated in patients with persistent heel pain and patients with unilateral lipoma of the calcaneus, and CT scans should be offered to rule out the involvement of the opposite side.
Sani et al. (2017) [8]	Intraosseous lipoma of the calcaneus: The non-stereotypic lesion of the bone	Entity is a very infrequent benign tumor-like lesion, mostly asymptomatic and discovered incidentally by imaging, including simple x-ray and CT/MRI. It has a good prognosis by conservative means and occasionally needs excision and curettage or bone grafting.
Kapukaya et al. (2006) [4]	Osseous lipoma: eleven new cases and review of the literature	Commented on involution of lesions and their detection and presentations at different bones and stages. Most patients healed with symptomatic treatment alone without surgery.

There are a number of modalities used for symptomatic relief, including analgesics, anti-inflammatory medications, sitz bath, soft sole or shoes, and temporary immobilization with silicon casts [8,10]. Surgical intervention in the form of curettage with or without bone grafting and augmentation with bone cement are indicated in painful, resistant cases or chronic cases, which are prone to pathological fracture or malignancy [2,5,9]. Most lesions are asymptomatic, diagnosed incidentally, and do not require surgery.

## Conclusions

This report describes a rare entity of an intraosseous lipoma of the calcaneum, which was initially treated as plantar fasciitis. Although intraosseous lipoma is a rare benign tumor, malignancy has been reported, so it needs to be evaluated properly. With the increasing use of MRI and CT scans, it is important to be aware of various differentials of chronic heel pain, including an intraosseous lipoma of the calcaneum.
